# Smart Polymers and Adaptive Systems in Pilot Suit Engineering: Toward Autonomous, Responsive, and Wearable Flight Technologies

**DOI:** 10.3390/nano15161228

**Published:** 2025-08-12

**Authors:** Hanjing Ma, Yuan He, Yu Ma, Guannan Han, Zhetao Zhang, Baohua Tian

**Affiliations:** 1 Apparel & Art Design College, Xi’an Polytechnic University, No. 19 Jinhua South Road, Xi’an 710048, China; mahanjing@xpu.edu.cn (H.M.); 20120710@xpu.edu.cn (Y.M.); 2 State Key Laboratory of Flexible Electronics (LoFE), Institute of Flexible Electronics (IFE), Northwestern Polytechnical University (NPU), 127 West Youyi Road, Xi’an 710072, China; 3 School of Arts, Xi’an International Studies University, Guodu Education and Technology Industrial Development Zone, Wenyuan South Road, Xi’an 710100, China; 107242023110044@xisu.edu.cn; 4 College of Design and Art, Shaanxi University of Science and Technology, Xi’an Weiyang University Park, Xi’an 710021, China; 2310zw025@sust.edu.cn

**Keywords:** nanomaterial and nanostructures, smart polymers, wearable electronics, on-body artificial intelligence, pilot suit systems

## Abstract

Next-generation pilot suits are evolving into intelligent, adaptive platforms that integrate advanced polymeric materials, smart textiles, and on-body artificial intelligence. High-performance polymers have advanced in mechanical strength, thermal regulation, and environmental resilience, with fabrication methods like electrospinning, weaving, and 3D/4D printing enabling structural versatility and sensor integration. In particular, functional nanomaterials and hierarchical nanostructures contribute critical properties such as conductivity, flexibility, and responsiveness, forming the foundation for miniaturized sensing and integrated electronics. The integration of flexible fiber-based electronics such as biosensors, strain sensors, and energy systems enables real-time monitoring of physiological and environmental conditions. Coupled with on-body AI for multimodal data processing, autonomous decision-making, and adaptive feedback, these systems enhance pilot safety while reducing cognitive load during flight. This review places a special focus on system-level integration, where polymers and nanomaterials serve as both structural and functional components in wearable technologies. By highlighting the role of nanostructured and functional materials within intelligent textiles, we underline a potential shift toward active human–machine interfaces in aerospace applications. Future trends and advancements in self-healing materials, neuromorphic computing, and dynamic textile systems will further elevate the capabilities of intelligent pilot suits. This review discusses interdisciplinary strategies for developing pilot wearables capable of responding to real-time physiological and operational needs.

## 1. Introduction

The rapid advancement of modern aviation technology has imposed unprecedented demands on pilot equipment [1,2]. Among them, pilot suits integrating cutting-edge interdisciplinary technologies such as high-performance fibers [3,4,5], advanced textile engineering [6,7,8,9], wearable devices [10,11,12,13], and electronic sensor integration [14,15,16] have garnered significant attention and research focus within the related industry and scientific community. In extreme high-altitude environments, pilots face challenges ranging from drastic temperature fluctuations and sustained gravitational forces up to 9 G to prolonged confinement in pressurized cockpits [17,18]. In addition to these operational demands, suits must ensure compliance with rigorous aerospace standards while providing resistance to fire resistance [19,20,21], tear [22,23], and high speed impact, particularly during emergency scenarios such as ejection or cabin failure. However, traditional flight suits offer only passive protection and fall short of the growing demand for intelligent functionalities such as real-time biosignal monitoring, adaptive thermal control, and anti-fatigue support, which are becoming increasingly critical under hypersonic conditions and extended mission durations.

In response to these limitations, advances in smart textiles [24,25,26,27], flexible electronics [28,29,30,31], wearable devices [32,33,34,35], and AI-powered signal analysis technologies [36,37,38,39] have greatly extended the functional scope of textile-based pilot suits. While numerous reviews have addressed smart textiles and wearable systems in consumer or biomedical contexts, their applicability to aviation-grade environments remains insufficiently discussed. This convergence is driving the evolution of next-generation aviation garments, enabling multifunctional integration through breakthroughs in materials and textile engineering. For instance, biomimetic thermal management fibers [40,41,42,43] and stretchable conductive fibers [44,45,46,47] now allow electronic components, such as epidermal electrodes, to be seamlessly embedded into fabrics, ensuring conductivity and functionality under dynamic flight conditions. High-performance fibers such as carbon fibers [48,49], aramid fibers [50,51,52], and ultrahigh molecular weight polyethylene (UHMWPE) fibers [53,54,55] offer superior strength-to-weight ratios and impact resilience, forming the mechanical backbone of modern flightwear.

**Figure 1:**
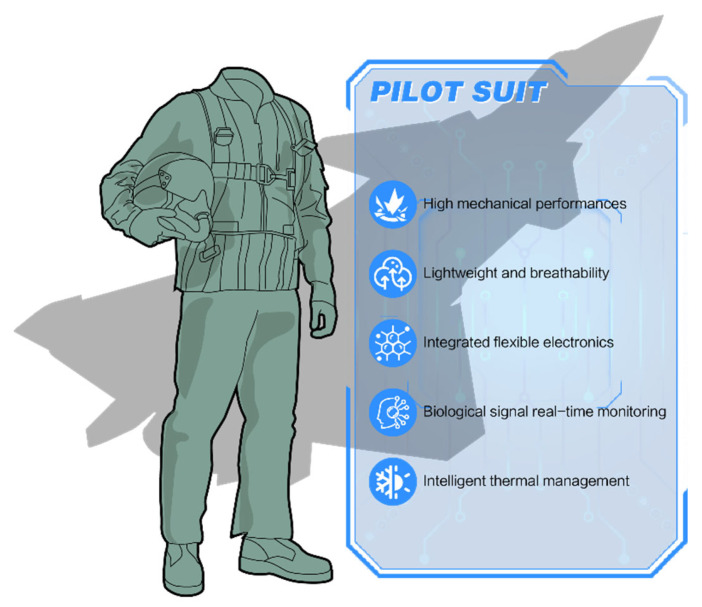
Next-generation integrated pilot suits: A multifunctional system combining high-strength composite materials, adaptive thermal regulation, and embedded biometric sensing modules.

However, integrating these innovations into mission-ready systems for aerospace applications presents persistent challenges. These include balancing thermal insulation and breathability, maintaining flexibility under repeated stress, and ensuring robust electronic integration without compromising fabric comfort or mobility. Solving these issues requires coordinated efforts across material science, nanotechnology, and systems engineering to achieve optimized performance across textile, mechanical, and electronic domains. The evolution of modern pilot suits demands a convergence of advanced materials, structural innovations, and intelligent integration to withstand extreme conditions while improving pilot safety and human–machine interaction (Figure 1). Among these, nanomaterials such as carbon nanotubes, MXenes, graphene, and nanoclusters have emerged as key enablers because of their ability to enhance strength, EMI shielding, and energy functions under the demanding conditions of aerospace environments [56,57]. When embedded into load-bearing fibers or multilayered fabrics, these nanoscale additives improve impact resistance, thermal regulation, and signal conductivity, supporting the development of multifunctional fibers for future flightwear.

Interdisciplinary collaboration is pivotal to overcoming limitations in conventional pilot suit technologies. Material scientists employ precise molecular engineering to develop gradient-structured fibers that integrate flame retardancy, freeze resistance, and electromagnetic shielding within a single textile platform [58,59,60,61]. Engineers have embedded aerogel composites and phase change materials into multilayered fabric systems, yielding ultralight suits that offer flame protection and thermal stability across broad temperature extremes [62,63,64]. Despite numerous reviews addressing these individual technologies, no existing work has comprehensively evaluated their convergence within the specific context of aviation-grade pilot suits, nor analyzed the integration challenges unique to this domain. In parallel, aerospace biomedical teams leverage finite element biomechanical models to optimize 3D-knitted compression zones, effectively reducing musculoskeletal strain during prolonged missions [65,66]. Together, these interdisciplinary efforts represent a shift from passive protection to intelligent, feedback-enabled systems. However, progress remains fragmented and lacks integrative analysis, highlighting the necessity for a comprehensive review that bridges nanomaterials, textile engineering, and flexible electronics.

This review responds to that need by systematically examining recent advancements and persistent challenges in developing high-performance smart pilot suits. Unlike prior reviews that primarily focus on consumer wearable devices or generalized smart textiles, this work emphasizes aviation-grade requirements, nanomaterials-driven functionalities, manufacturing strategies, and aerospace certification challenges. The discussion also addresses advanced 3D/4D manufacturing approaches and strategies for integrating sensors, energy modules, and intelligent feedback mechanisms. By aligning technological innovations with the complex demands of aviation missions, this work outlines a roadmap for intelligent pilot suits, addressing key barriers like material-device compatibility and fabrication scalability. The continued evolution of smart flightwear will rely on advances in interdisciplinary design, scalable production techniques, and the establishment of cohesive industry standards.

## 2. Desired Properties and Requirements for Modern Pilot Suits

### 2.1. Nanomaterials-Enhanced Textile Platforms for Pilot Suits

The integration of nanomaterials into textile substrates represents a pivotal strategy for enhancing the mechanical, thermal, and electrical performance of next-generation pilot suits. Nanomaterials exhibit exceptional intrinsic properties at the nanoscale, such as high strength-to-weight ratios, large specific surface areas, outstanding electrical conductivity, and tunable surface chemistries [67,68]. These unique features enable multifunctionality beyond traditional textile capabilities and address critical aviation requirements, including structural reinforcement, adaptive thermal management, real-time sensing, and electromagnetic interference (EMI) shielding [69,70]. Structure–property relationships play a decisive role in these applications. For example, carbon nanotubes (CNTs) and graphene create highly interconnected conductive pathways within fiber matrices, resulting in stable signal transmission, strain-sensitive response under deformation, and enhanced load distribution during mechanical stress [71,72]. Their inherent flexibility also supports integration without compromising garment comfort. MXenes, with their two-dimensional layered structures and surface terminations, provide high electrical conductivity, EMI attenuation, and redox-active sites suitable for energy storage components embedded in flight suits [73]. Their ability to accommodate ion transport also supports electrochemical sensors and energy modules. Metal nanoclusters (e.g., Ag nanoclusters) introduce catalytic reactivity and fluorescence properties, enabling dynamic biosignal sensing or self-diagnostic functionality within textile-integrated systems [74,75]. Metal–organic frameworks (MOFs) contribute high porosity and tunable chemistry for gas sorption, thermal buffering, and environmental monitoring [76,77], which are relevant for cockpit or high-altitude conditions.

To bridge laboratory innovation with aviation relevance, these nanomaterials are typically processed through scalable strategies such as dip-coating, wet spinning, inkjet printing, and electrospinning, which allow their integration into fibers, yarns, or multilayer textile structures [78,79]. While these methods demonstrate promising performance in wearable and protective textiles, their adaptation to aviation-grade pilot suits requires further validation in terms of durability under cyclic thermal loads, resistance to high-altitude humidity, and compliance with flammability and EMI-shielding standards. Table 1 summarizes representative nanomaterials, their functional contributions, and integration strategies within fiber-based systems relevant to aerospace applications. These examples illustrate how nanoscale design principles can be translated into textile-level functionalities for future pilot suits, while also highlighting the need for systematic assessment of long-term reliability and regulatory compliance.

### 2.2. High-Performances Fibers for Modern Pilot Suits

**Figure 2:**
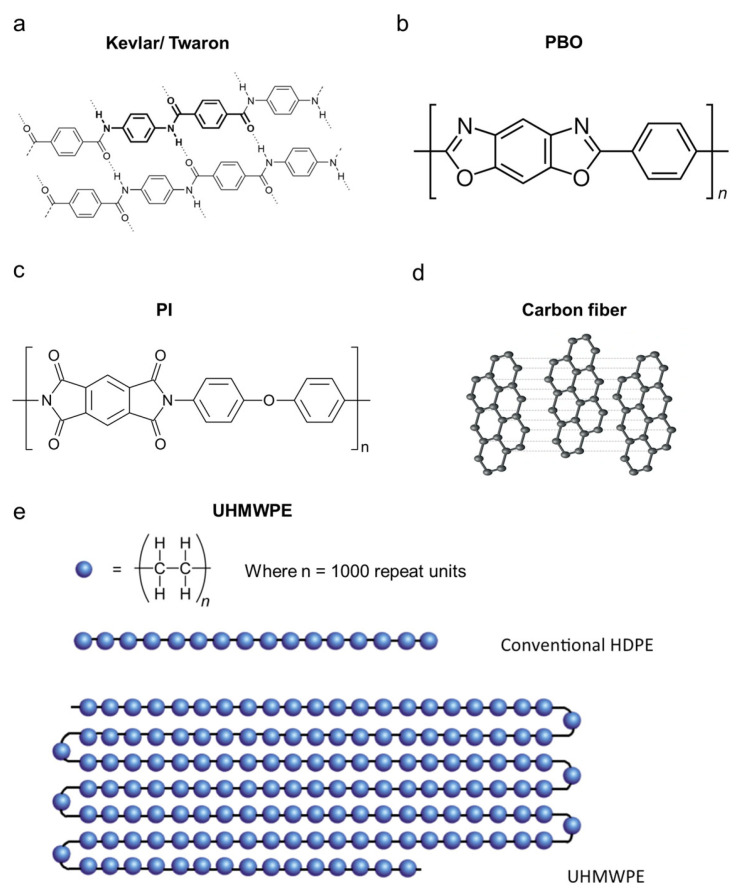
Typical top engineering polymeric fibers and their molecular structures for advanced pilot suits. (**a**) Kevlar and Twaron: aromatic polyamide chains with strong hydrogen bonding and π–π stacking for strength and flame resistance. (**b**) PBO: rigid benzobisoxazole units offering exceptional thermal stability. (**c**) Polyimide: imide ring structure enabling wide thermal tolerance. (**d**) Carbon fiber: graphitic stacking providing strength, thermal, and electrical conductivity. (**e**) UHMWPE: highly oriented polyethylene chains delivering ultrahigh tensile strength, low density, and excellent abrasion resistance.

To meet the extreme demands of modern pilot suits, a class of high-performance engineering fibers such as aramid fibers including Kevlar^®^ (DuPont, Wilmington, DE, USA) and Twaron^®^ (Teijin Aramid, Arnhem, The Netherlands), poly(p-phenylene-2,6-benzobisoxazole (PBO), polyimide (PI), carbon fiber, and ultrahigh molecular weight polyethylene (UHMWPE) has been developed. These five top engineering polymeric fibers are widely utilized in environments involving extreme conditions such as high altitude and large temperature fluctuations; therefore, they also hold strong potential for application in advanced pilot suits. Their exceptional mechanical strength, flame resistance, and thermal stability arise from distinct molecular and nanoscale architectures. Aramid fibers consist of aromatic polyamide chains with strong hydrogen bonding and π–π stacking, forming crystalline domains that provide high tensile strength and self-extinguishing behavior (Figure 2a), resulting in excellent tensile strength and flame retardancy, which are prioritized for their unparalleled strength-to-weight ratio (3.6–4.1 GPa tensile strength at 1.44 g/cm^3^) [85,86]. PBO (Zylon^®^, Toyobo Co., Ltd., Osaka, Japan) possesses a rigid heterocyclic backbone, producing ultrahigh modulus and thermal resistance [87], making it suitable for applications exposed to high heat (Figure 2b). PI fibers contain imide ring aromatic backbones, granting them broad thermal tolerance and flame resistance, ideal for thermal insulation layers (Figure 2c). For example, PI fibers offer broad temperature functionality (−269 °C to 300 °C) and moderate flame resistance with a limiting oxygen index (LOI) of 38–42, serving as thermal insulators in multilayer suits [88,89]. Carbon fibers are composed of aligned graphitic (sp^2^) sheets, delivering ultrahigh stiffness, conductivity, and fatigue resistance, essential for structural and electronic integration (Figure 2d). Carbon fibers are composed of aligned graphitic (sp^2^) sheets, delivering ultrahigh stiffness, conductivity, and fatigue resistance, essential for structural and electronic integration (Figure 2d). UHMWPE fibers consist of highly oriented polyethylene chains (Figure 2e), providing exceptional tensile strength, very low density, and excellent abrasion resistance [53]. These properties make UHMWPE an ideal candidate for lightweight, impact-resistant applications in advanced pilot suits.

Despite their compositional diversity, many of these fibers share similar synthesis principles, generally involving solution or melt polymerization followed by high-temperature drawing or stabilization. For example, aramids and polyimides are produced via step-growth polycondensation followed by dry-jet wet spinning [90]. Carbon fibers are derived from polymeric precursors such as polyacrylonitrile (PAN) or pitch through controlled carbonization and graphitization [91]. These processes promote high crystallinity and chain orientation, which directly influence their tensile performance, thermal resistance, and fatigue durability. Table 2 summarizes typical engineering fibers and their key performance metrics relevant to pilot suit applications. These nanoscale features such as high crystallinity, chain alignment, and structural rigidity translate into textile-level performance, enabling lightweight, durable, and multifunctional flightwear. They also provide a foundation for nanomaterial integration and smart textile systems discussed in later sections.

### 2.3. Thermal-Regulative Fibers for Modern Pilot Suits

Effective thermal regulation is vital for maintaining pilot physiological stability and mission effectiveness under extreme thermal conditions. As written in the paper by Haishen et al., ambient temperatures at cruising altitude can fall as low as −65 °C, creating severe cold stress for pilots [96]. Conversely, as reported by Shetty et al., cockpit temperatures may even rise more than 45 °C above the ambient environment due to solar radiation and avionics heat load, resulting in heat-saturated conditions [17]. Sustained thermal stress under these extremes can impair cognitive function, motor coordination, and cardiovascular performance, thereby elevating the risk of operational failure during high gravitational force (high-G) maneuvers or emergency scenarios.

Effective thermal regulation is vital for maintaining pilot physiological stability and mission effectiveness in the face of extreme ambient fluctuations [97]. Sustained thermal stress can impair cognitive function, motor coordination, and cardiovascular performance, thereby elevating the risk of failure during high-G maneuvers or emergency operations [98].

**Figure 3:**
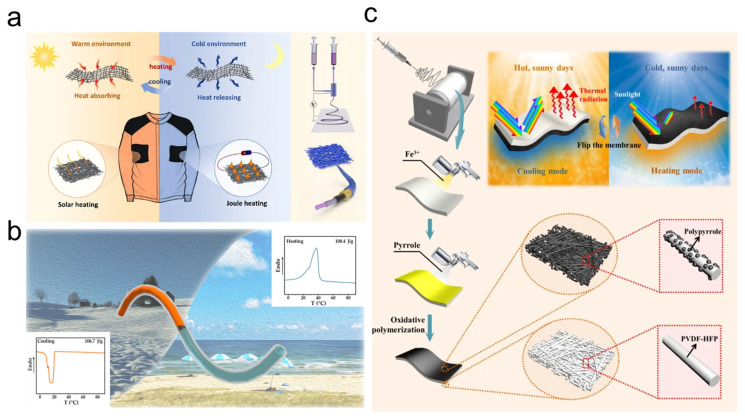
Multifunctional fiber architecture for next-generation pilot suit thermal regulations. (**a**) Multilayer core–sheath architecture enabling environmental-responsive thermal regulation through tunable infrared emissivity and conductive heat pathways [41]. (**b**) Microfluidic-encapsulated PCM fibers providing mission duration latent heat storage/release via pressure suit-integrated phase change networks [100]. (**c**) Dual-mode radiative membranes achieving sunlight thermal management through solar-reflective/infrared-absorptive surface modulation for cockpit-adjacent thermal buffering [105].

An integrated thermal management solution for modern pilot suits that synergizes passive insulation with active regulation through multilayer fiber technologies was proposed by combining aerogel-enhanced ceramic composites for extreme environmental protection, phase change polymer fibers for physiological heat buffering, and graphene-enabled electroresponsive textiles that dynamically adjust thermal conductivity (Figure 3a). The system employs microfluidic-encapsulated phase change materials to stabilize core body temperatures during rapid altitude changes. Meanwhile, conductive networks autonomously modulate ventilation porosity and heat dissipation in response to real-time biometric feedback. This enables dynamic thermal management driven at the molecular level by reversible hydrogen bonding and hydrophobic rearrangements within the polymer matrix [99]. Together, these features help create an adaptive thermal microenvironment that maintains pilot comfort across stratospheric cold and engine-adjacent heat extremes, supported by continuous energy harvesting from integrated solar panels and latent heat redistribution mechanisms. Meanwhile, smart materials help stabilize core body temperatures during rapid altitude changes. Conductive networks autonomously modulate ventilation porosity and heat dissipation in response to real-time biometric feedback. This supports dynamic thermal management driven at the molecular level by reversible hydrogen bonding and hydrophobic rearrangements within the polymer matrix, which facilitates creating an adaptive thermal microenvironment that maintains pilot comfort across stratospheric cold and engine-adjacent heat extremes through continuous energy harvesting from integrated solar panels and latent heat redistribution mechanisms [41].

Other studies have revealed a phase change composite fibers for next-gen pilot thermal suits by microfluidic encapsulation of polyethylene glycol 1000 (PEG1000) within hollow polypropylene fibers, achieving significant encapsulation efficiency [100]. These flexible fibers maintain 94% latent heat capacity versus pure PEG1000 while demonstrating mechanical durability through 50 plus thermal cycles, enabling their integration into pressure suit liners as passive thermal buffers (Figure 3b). Such reversible phase transitions are governed not only by bulk enthalpic changes but also by microphase behavior at the molecular level, particularly the cooperative interplay between PEG’s hydration shell and dynamic hydrogen bonding [99]. The core–sheath architecture preserves PEG’s phase change reversibility and elevates its decomposition temperature by 8 °C compared to the bulk form, enabling stable thermal buffering across extremely low to high cockpit temperatures. In synergy with active electrocaloric systems, this design enhances adaptive thermal regulation under fluctuating flight conditions [101]. Such dual-mode control contributes to up to 40% energy savings in aviation life support systems via diurnal heat banking, while the moisture-resistant PP matrix improves long-term durability and mitigates performance degradation under cyclic thermal loads [102].

However, limitations remain for these phase change composite fibers. The encapsulated PEG1000 may suffer from leakage or performance degradation under long-term mechanical stress or extreme thermal cycling, particularly if the encapsulation shell integrity is compromised [103]. Additionally, the low thermal conductivity of polypropylene can delay heat exchange efficiency, and the relatively narrow phase change temperature window of PEG1000 limits its adaptability to rapidly fluctuating cockpit environments [104].

On the other hand, an innovative dual-mode thermal regulation system is reported, which is also attractive for next-generation pilot suits [105]. This system is constructed through hierarchically structured polymer membranes combining radiation modulation and solar energy conversion (Figure 3c). By integrating poly(vinylidene fluoride-co-hexafluoropropene) and polypyrrole fibers, the system achieves bidirectional environmental adaptation while simultaneously reflecting solar radiation, which is superior for cockpit-adjacent cooling when harnessing infrared wavelengths for cabin-independent heating.

Building on these individual strategies, future research should explore their synergy within multiple thermal management systems. Combining phase change fibers, radiative membranes, and multilayer conductive architectures could yield pilot suits that autonomously adapt to extreme environmental gradients without external power input. We envision that such hybrid platforms will leverage nanoscale engineering, smart coatings, and responsive polymers to achieve real-time thermal modulation. Ultimately, this convergence of technologies has the potential to transform pilot from passive protective layers into intelligent systems capable of stabilizing body temperature during abrupt cockpit temperature transitions while ensuring operational comfort and safety.

### 2.4. Energy-Harvesting Triboelectric Fibers for Modern Pilot Suit

Energy-harvesting triboelectric fibers offer a practical solution for next-generation pilot suits by enabling self-sustained power generation from pilots’ natural movements, such as limb adjustments during flight operations, addressing the growing demand for autonomous power systems [106,107,108]. This strategy reduces battery dependency, as TENG systems inherently function like capacitors rather than batteries due to their electrostatic charge separation mechanism, offering high voltage but low current output typical of capacitive devices, and are thus often coupled with supercapacitors or small rechargeable cells to stabilize energy delivery and extend operational time [109,110].

Advances in nanotextured polymer composites have improved charge transfer efficiency, making power output feasible even within pressurized suits [110]. These enhancements originate from the triboelectric effect, where electron transfer occurs upon periodic contact and separation between two dissimilar materials [111]. Nanostructured features such as nanopillars, nanowires, and hierarchical roughness increase the real contact area and local electric field, thereby amplifying surface charge density. Additionally, these nanostructures facilitate electrostatic induction, which further boosts charge accumulation during sliding or bending motions [112]. These designs typically incorporate conductive nanofillers such as carbon nanotubes and MXene nanosheets to create interconnected conductive pathways and high-permittivity interfaces, which amplify local electric fields and reduce internal impedance. This structural modification promotes efficient electron transfer and charge accumulation during contact electrification and electrostatic induction, thereby enhancing overall energy conversion performance [113,114]. This nanofiller integration not only boosts surface charge density but also supports hybrid electrostatic-capacitive energy storage by reducing internal impedance and improving charge retention stability.

**Figure 4:**
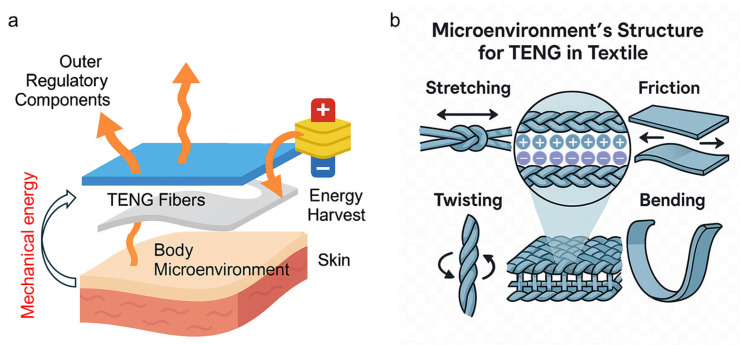
Schematic illustration of the microenvironment within a modern pilot suit. (**a**) Mechanical interactions between the human body and embedded smart textile layers provide potential for energy harvesting. Arrows indicate heat or energy flow paths. (**b**) Movements such as stretching, bending, torsion, and friction between fibers and skin generate triboelectric signals, enabling self-powered functionality within wearable systems.

Concerning modern pilot suits, design strategies can draw inspiration from concepts discussed in wearable systems, such as fabrics integrated with triboelectric nanogenerator (TENG) fibers that leverage the inherent advantages of textile materials as energy harvesting media. Smart fabrics, functioning as the body’s closest environmental interface, inherently possess a large surface area and intimate contact with the skin, enabling efficient capture of both mechanical energy from limb movements and thermal gradients between the body and flight suit microenvironment [115,116]. The mechanical interactions between the human body and embedded textile layers (Figure 4a), as well as movements such as stretching, bending, torsion, and friction between fibers and skin (Figure 4b), play essential roles in facilitating triboelectric signal generation.

In addition, Table 3 summarizes representative energy outputs under different pilot motion scenarios and the structural compatibility of TENG fibers with layered suit architectures. Integration feasibility was evaluated based on parameters including fabric layer adhesion, electrical interfacing with onboard electronics, and mechanical compliance under dynamic strain. These assessments demonstrate that triboelectric fibers can be effectively embedded into current multilayered pilot suit systems without compromising mobility, thermal balance, or environmental shielding.

**Figure 5:**
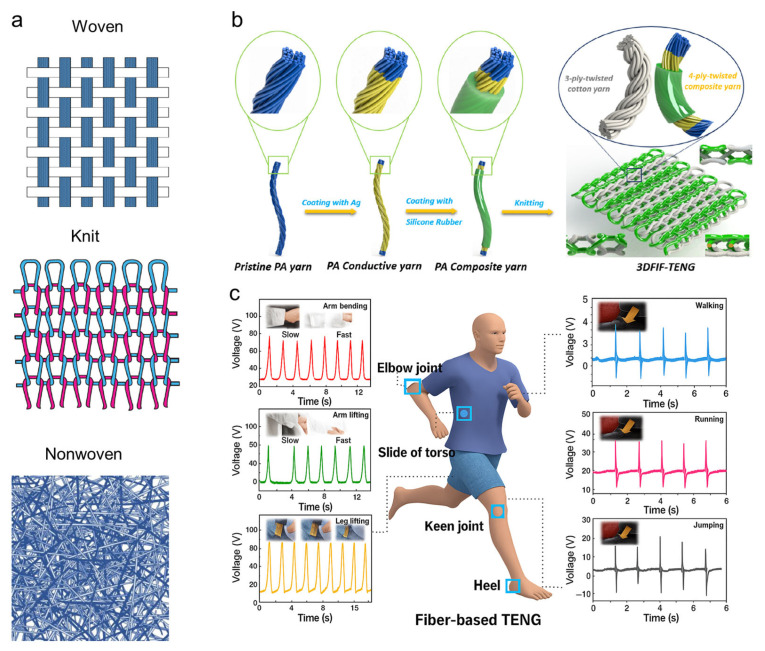
Energy-harvesting triboelectric fibers for pilot suit smart textiles. (**a**) Textile architectures: Woven (interlaced yarns with blue color indicating warp yarn and white color indicating weft yarn), knitted (flexible loops), and nonwoven (random fibers distributed without specific direction) optimized for mechanical energy harvesting [123]. (**b**) 3D-TENG fabric: Silver/silicone-coated PA66 conductive yarns interlocked with dielectric cotton yarns [124]. (**c**) Self-powered motion sensor based on dopamine-modified MXene/thermoplastic polyurethane (TPU) composite fiber for joint motion tracking. The sensor exhibits a peak voltage of ~0.85 V under 60° bending with a response time of ~85 ms and stable performance over 1500 cycles. Distinct voltage outputs during walking (~0.42 V), running (~0.64 V), and jumping (~0.87 V) demonstrate effective motion discrimination [125].

For example, their structural adaptability to complex deformations is achieved through engineered woven, knitted, and nonwoven architectures (Figure 5a), which maintain dynamic conformity to bodily motions while preserving triboelectric performance [123]. The three-dimensional textile configuration synergizes conductive yarns with dielectric substrates, capitalizing on fabrics’ intrinsic properties of flexibility, breathability, and stretchability (Figure 5b) [124]. The introduced 3D double-faced interlock fabric triboelectric nanogenerator (3DFIF-TENG) is fabricated using silver-coated polyamide conductive yarn, silicone rubber, and silicone elastomer base, which is developed through a customized double needle bed flat knitting technology, offering textile-based solutions to overcome limitations of conventional wearable devices. Moreover, advanced manufacturing techniques like embroidery allow seamless integration of MXene-enhanced functional fibers into existing textile matrices, creating an unobtrusive energy-harvesting platform that simultaneously powers cockpit instruments and monitors physiological signals (Figure 5c) [125]. Such similar integration has been demonstrated to maintain mechanical flexibility and enhance signal responsiveness in smart garments designed for aerospace and military applications. Moreover, this textile–electronics convergence retains wearer comfort while achieving dual functionality, i.e., autonomous energy generation from pilot movements (control inputs, ejection sequences) and real-time health monitoring via triboelectric signal patterns. Several recent studies have further supported the potential of MXene or nanomaterial-based fiber systems for dual energy sensing and harvesting [126,127].

Despite their optimal mechanical, thermal, and functional properties, it is important to note that many advanced materials discussed in this section such as MXene-coated fibers, PBO, shape memory polymers, and other smart textile systems have not yet achieved formal aviation-grade certification. These materials often remain at the research or prototyping stage, and their performance under long-term operational stressors such as UV radiation, high-altitude thermal cycling, moisture ingress, and outgassing has yet to be validated according to established aerospace standards (e.g., RTCA DO-160, MIL-STD-810) [128,129]. Nevertheless, their integration potential within pilot suit systems, particularly in wearable sensing, thermal regulation, and structural reinforcement, indicate that they can serve as next-generation candidates for aerospace adoption. With further development, testing, and regulatory engagement, these materials gradually advance toward qualification for flight applications as their performance advantages become more widely recognized in defense and commercial aviation sectors.

## 3. Advanced Manufacturing Techniques for Potential Modern Pilot Suits

As aviation technology evolves rapidly, the demands placed on pilot suits are also advancing beyond traditional materials and textile industry [130]. Next-generation pilot suits are required to offer not only superior protection but also adaptive functionality, optimized ergonomics, and enhanced integration with onboard systems [131]. To meet these challenges, suits must incorporate materials with nanoscale structural design such as hierarchical fiber composites and responsive interfaces that enable functional responsiveness to physiological changes, environmental stimuli (e.g., temperature, pressure), and mission-specific requirements through localized energy conversion, heat regulation, or signal transduction [123,132]. To support these evolving requirements, manufacturers are turning to cutting-edge production methods such as additive manufacturing, digital textile engineering, and composite layering, which allow the creation of lightweight, responsive, and highly customizable gear [133]. These advanced techniques not only improve the functional performance of the suits but also streamline development, reduce material waste, and open the door to rapid prototyping and field-ready customization.

### 3.1. Three-Dimensional Manufacturing

Additive manufacturing, particularly through 3D-warping, 3D-printing technologies, is reshaping the development landscape of modern pilot suits by enabling the fabrication of complex, lightweight, and highly customizable components [134]. Unlike traditional manufacturing methods, which often involve cutting, stitching, or layering materials manually, additive manufacturing builds components layer by layer, allowing for intricate geometries and seamless integration of functional elements [135].

**Figure 6:**
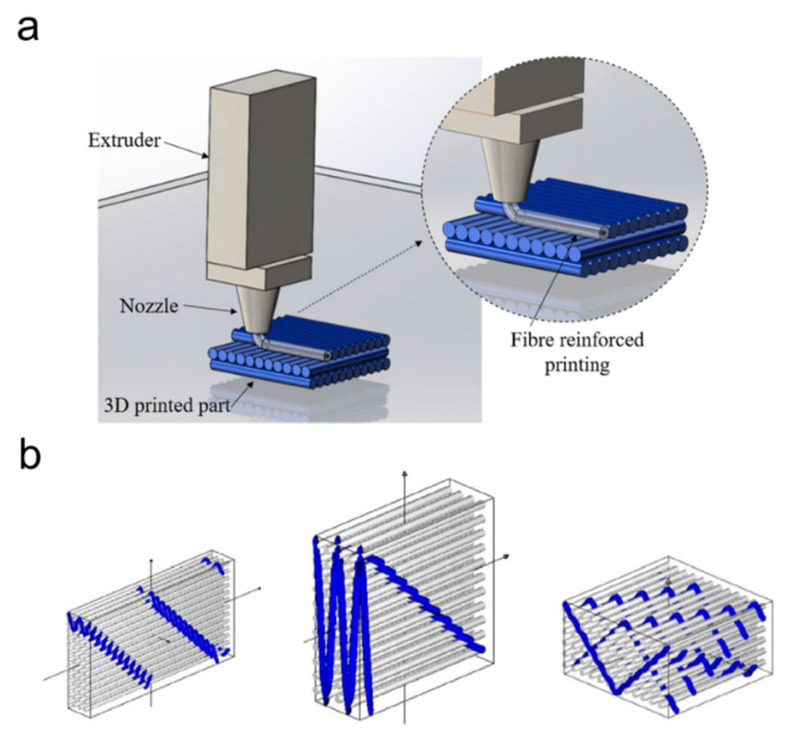
Three-dimensional manufacturing techniques. (**a**) Additive manufacturing by 3D printing, capable of producing personalized anatomical structures with a resolution down to 50–100 μm and mechanical strength up to 15 MPa [136]. (**b**) 3D warp interlock fabrics manufactured by warp interlock weaving, which provide high formability and ballistic energy absorption. In ballistic testing, a panel composed of 40 layers of 3D warp interlock fabric absorbed up to 97% of the projectile energy, with maximum backface signature depths ranging from 23 to 36 mm [140]. Blue colored line indicates the printed fiber injected onto the layer or the interlocking fiber within the woven fabric.

To be specific, 3D fiber-reinforced printing is a typical advanced additive manufacturing technique that embeds strong fibers like carbon or Kevlar into thermoplastic materials, significantly enhancing strength and durability (Figure 6a) [136]. This process works by combining layer-by-layer thermoplastic deposition with in situ placement of continuous fibers, which are aligned along selected directions during printing to improve load-bearing capacity [137]. In contrast to conventional 3D printing methods that rely solely on thermoplastic injecting, this technique introduces continuous fibers during the layer-by-layer deposition process, aligning them parallel to one direction. Such parallel fiber integration increases tensile performances along this direction and enhances interlayer bonding strength, thereby improving resistance to shear failure [138]. The reinforcement effect originates from fiber matrix composing, where the polymer provides shape adaptability and the embedded fibers deliver high tensile and flexural strength, significantly reducing interlayer delamination [139].

In modern pilot suits, this technology enables the creation of lightweight, high-performance components that withstand extreme conditions, creating custom-fit exoskeletal supports that enhance joint mobility and reduce pilot fatigue during high-G maneuvers. Additionally, it allows for precise customization to individual body shapes, improving comfort and mobility.

However, due to the gravitational effect during layer-by-layer fabrication, support structures are typically required for overhangs and complex geometries, which adds to the post-processing burden. Compared to traditional methods, 3D printing offers three key advantages: (1) geometric freedom for complex structures, (2) reduced material waste, and (3) rapid prototyping with shorter production cycles. On the other hand, its main limitations include (1) lower interlayer strength relative to bulk materials, (2) limited availability of high-performance flexible materials suitable for wearables, and (3) the need for post-processing to remove supports and improve surface finish. These trade-offs must be considered when applying 3D printing technologies to flight garments.

On the other hand, 3D warp interlock fabrics are innovative textile structures increasingly used as fibrous reinforcements that can also be utilized in modern pilot suits due to their superior performance in demanding aerospace environments (Figure 6b) [140]. Unlike conventional 2D woven fabrics, 3D warp interlock fabrics incorporate yarns in the warp, weft, and Z-direction (through-thickness), creating a highly integrated structure that offers enhanced delamination resistance, greater dimensional stability, and improved load distribution [141]. These fabrics are typically made from high-performance fibers such as aramid (e.g., Kevlar^®^), carbon, or PBO, which provide excellent flame resistance, high tensile strength, and low thermal conductivity, which are essential properties for protection against high temperatures, fire hazards, and extreme gravitational forces [142].

### 3.2. Four-Dimensional Manufacturing

Four-dimensional printing represents an advancement in additive manufacturing, enabling materials to change shape or properties over time in response to external stimuli such as heat, light, moisture, or magnetic fields [9,143]. For modern pilot suits, especially those used in high-performance aviation or space operations, 4D printing offers unique opportunities for enhancing adaptability, comfort, and safety. For instance, 4D printing builds upon traditional 3D printing by incorporating smart materials such as shape memory polymers (SMPs), hydrogels and liquid crystal elastomers into the printing process. At the nanoscale, SMPs contain crosslinked networks with switching segments that store elastic energy, enabling reversible shape fixation and recovery when heated [144]. This feature allows suit components to adapt fit or stiffness under cockpit temperature variations. Hydrogels possess hydrophilic polymer networks with nanoscale porosity and hydrogen bonding, facilitating water uptake and humidity-driven swelling for passive cooling [145]. LCEs (liquid crystal elastomers) consist of aligned mesogenic domains that reorient under thermal or optical stimuli, producing reversible anisotropic deformation [146]. These nanostructural properties make SMPs ideal for thermally adaptive panels, hydrogels for breathable moisture-regulating layers, and LCEs for localized flexibility, which are critical for maintaining comfort and performance under extreme aviation conditions, making them highly suitable for pilot suit applications requiring thermal responsiveness, breathable comfort, and movement adaptability [147,148]. In the context of pilot suits, this translates into garments that adjust their fit, thermal insulation, or structural rigidity dynamically, depending on altitude, temperature, or gravitational forces.

One prominent 4D printing technique for pilot suits is multi-material direct ink writing (DIW). This method allows the precise placement of different responsive materials in a single printing process. For instance, an area around the spine is printed with a stiff SMP to provide impact protection, while sections near joints might use a more elastic, breathable material that contracts or expands with the pilot’s movement or body heat.

**Figure 7:**
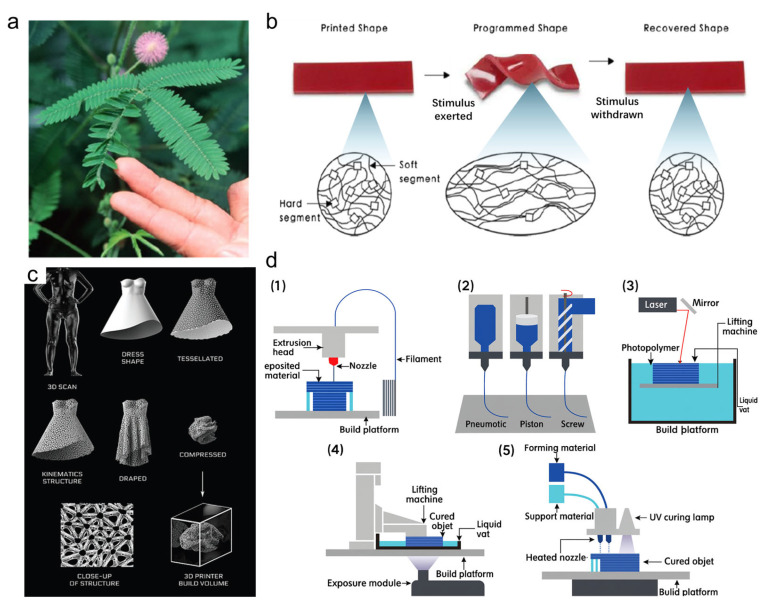
Inspiration, mechanism, applications, and fabrication strategies for 4D printing on textiles. (**a**) Mimosa-inspired 4D printing materials [149]. (**b**) Molecular mechanism of thermally activated shape memory polymers with dual-shape capability [151]. (**c**) Mathematical and geometric modeling used to create simulated designs [8]. (**d**) Technical schematic illustrating the 4D printing of smart textiles using various printing technologies: (**d1**) Fused Deposition Modeling (FDM) for thermoplastic components; (**d2**) Direct Ink Writing (DIW) for flexible, shape-shifting designs; (**d3**) Stereolithography (SLA) for complex resin curing; (**d4**) Digital Light Processing (DLP) for photopolymer curing; (**d5**) PolyJet Printing for multi-material components [152].

The earliest inspiration for 4D printing materials came from Mimosa pudica [149], a plant known for its rapid and reversible movements in response to external stimuli such as touch or force (Figure 7a). This sensitive plant exhibits a complex internal structure that enables it to fold its leaves quickly when disturbed. Scientists and engineers have drawn from this natural behavior to design smart materials that can change shape over time or under specific conditions. For insurance, bilayer structures composed of hydrogel-based polymers such as poly(N-isopropylacrylamide) (PNIPAm) can be utilized, exhibiting a lower critical solution temperature (LCST) phase transition. Upon heating above LCST (~32 °C), PNIPAm becomes hydrophobic and contracts, while the passive layer remains unchanged, creating differential stress that causes bending or folding. This behavior mimics the nastic motion of Mimosa pudica and serves as a key mechanism behind 4D-printed shape reconfiguration [150]. These biomimetic materials form the foundation of 4D printing, where printed objects are programmed to transform in response to environmental triggers like heat, moisture, or mechanical force. Shape memory polymers (SMPs) are core materials in 4D printing (Figure 7b) [151]. Based on phase transition mechanisms, they can be integrated with advanced textiles to create functions beyond those of conventional 3D-printed fabrics. Under specific critical conditions, these composites undergo significant structural transformations. This unique property enables smart responses to environmental changes. For instance, in aerospace applications, SMP-based 4D-printed fabrics can be used in pilot suits, providing adaptive protection or activating special features in extreme or sudden conditions. Such innovations improve safety and functionality, supporting the development of wearable technologies for demanding environments. Mathematical and geometric modeling plays a crucial role in designing functional 4D-printed textiles (Figure 7c) [8]. As highlighted, such modeling integrates differential geometry and finite element simulations to predict how shape memory textiles will deform in response to external stimuli such as heat or moisture. Specifically, body surface scanning data such as curvature, joint constraints, and strain localization can be computationally converted into input parameters for smart textile architectures. This allows engineers to pre-program actuation paths into printed textiles, ensuring functional performance and shape reconfigurability under mission-specific conditions. Building on this model-informed design process, this data is then used to generate customized digital models that guide the placement and orientation of 4D printing materials. Strain-hardening polymers can be strategically embedded into critical areas such as the elbows, knees, and neck through 4D printing techniques. These polymers can undergo significant structural transformation when exposed to external stimuli such as heat or mechanical impact. Under sudden events like high temperatures or collisions, the material stiffens or reshapes to offer localized protection. This simulation-driven, customized design approach enables garments that not only fit precisely but also respond intelligently to extreme conditions. By integrating mathematical modeling with responsive material science, this process represents a major advancement over traditional textile engineering, allowing the creation of protective, adaptive garments tailored for high-risk applications like aviation or space exploration. Currently available 4D printing technologies for smart textiles offer versatility in materials, precision, and functionality, enabling the creation of adaptive, responsive fabric structures. These techniques include Figure 7(d1): Fused Deposition Modeling (FDM), where filamentary material is melted and extruded layer by layer, with auxiliary structures printed to support subsequent layers; Figure 7(d2): Direct Ink Writing (DIW), which extrudes ink that forms shapes based on its rheological properties, suitable for printing flexible microstructures; Figure 7(d3): Stereolithography (SLA), utilizing a light source to cure photosensitive materials layer by layer, ideal for complex shapes; Figure 7(d4): Digital Light Processing (DLP), which uses a digital light projector to cure photopolymers efficiently with each layer exposed in a single step; and Figure 7(d5): PolyJet Printing, combining liquid print molding and light-curing resin to produce high-quality components with minimal step effects [152]. These methods enable the integration of time-dependent responses, mechanical adaptability, and environmental sensitivity, supporting advanced applications in aerospace, protective clothing, and wearable healthcare devices.

Table 4 summarizes the key characteristics of advanced 4D-printable polymers suitable for pilot suit applications, including their chemical composition, responsive stimuli, functional benefits, integration challenges, printing compatibility, and mechanical performance, providing a comparative overview to guide material selection for smart, adaptive, and protective textile systems. Overall, 4D printing manufacturing techniques for pilot suits enable the development of intelligent, responsive garments that actively adapt to the pilot’s environment and physical condition. Through precise deposition of smart materials and integration of dynamic design features, these suits promise a new standard in functionality and protection for aerospace and military applications.

In addition to passive responsiveness, the integration of active actuation and closed-loop feedback systems can enhance the adaptability of smart pilot suits for high-altitude and extreme-flight environments. For instance, programmable actuators based on electrothermal, pneumatic, hydrogel expansion, shape memory alloys, or electroactive polymers will dynamically adjust garment compression, ventilation, insulation, structural posture, or muscle support in response to real-time physiological inputs such as heart rate, body temperature, or gravitational force exposure. Coupled with embedded sensor networks, these systems enable autonomous feedback loops, allowing the suit to modulate its functional state without pilot intervention. Although challenges remain in miniaturizing these actuators and ensuring compatibility with aerospace constraints (e.g., weight, latency, power supply), foundational elements for responsive systems are under development that support future mission-adaptive pilot suits. Table 5 summarizes representative actuation mechanisms and their potential integration into future aerospace suit architectures.

## 4. System Integration of Flexible Electronics in Pilot Suits

The integration of flexible electronics into textile or clothes represents a critical step toward realizing fully functional, intelligent garments capable of real-time sensing, communication, and adaptive response [170]. While advanced fibers and manufacturing techniques provide the foundational materials and structures, true utility arises from how these elements are assembled into cohesive and interoperable systems [171]. System integration refers to the strategic embedding of sensors, circuits, interconnects, power supplies, and communication modules within the textile framework in a way that maintains comfort, flexibility, and durability under extreme operational conditions [172]. Successful integration must address several challenges unique to the wearable environment such as mechanical deformation, sweat and moisture exposure, and electromagnetic interference while ensuring reliable signal transmission and power efficiency [173]. For pilot applications, these systems must also be lightweight, ergonomic, and non-intrusive, minimizing any impediment to the pilot’s performance. This section explores various integration strategies, including the seamless incorporation of fiber-based electronics, modular and hybrid approaches, and the use of stretchable or self-healing interconnects. Additionally, we examine technologies for wireless data transmission and on-body power management, both of which are essential for autonomous operation in dynamic aerospace settings. Collectively, these innovations bring us closer to the next generation of responsive, high-performance pilot suits.

### 4.1. Integration Strategies for Fiber-Based Sensors and Circuits

**Figure 8:**
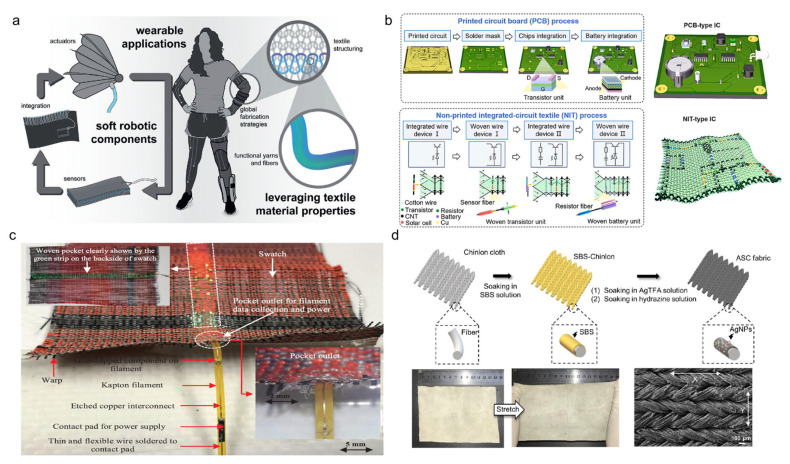
Candidate integration strategies for modern pilot suit assembly. (**a**) Functional components and performance metrics of robotic garments, with textile structures for sensor and actuator integration [10]. (**b**) Comparison of traditional PCB-based ICs with non-printed integrated-circuit textile (NIT) processes and NIT-type ICs [175]. (**c**) LED yarn with two-terminal connection embedded in a flexible textile circuit for lighting or signaling use [176]. (**d**) Fabrication scheme of ASC fabric (A: AgNPs, S: SBS, C: Chinlon), with pre-/post-stretch images and SEM at varying magnifications [177].

Integrating sensors and circuits into textiles requires innovative approaches to maintain flexibility and durability. Recent relative studies have explored embedding sensors directly into fibers, allowing for seamless integration into garments [174]. These fiber-based sensors can monitor various physiological parameters, enhancing the functionality of textile-based clothes or wearables [172]. Building on the system-level perspective outlined in Figure 8a, the integration of fiber-based sensors is essential to enabling multifunctional and responsive capabilities with potentials in modern pilot suits [10]. These sensors allow real-time monitoring of physiological and environmental parameters such as heart rate, temperature, motion, and cabin conditions without compromising comfort or mobility.

Figure 8b contrasts traditional printed circuit board (PCB) integration with emerging non-printed integrated-circuit textile (NIT) technologies, both critical considerations for modern pilot suits [175]. Traditional PCBs are rigid and bulky, typically housed in protective casings and attached as modules. While reliable, their inflexibility limits seamless integration into garments, adding weight and reducing comfort, which is not ideal for pilot suits requiring full mobility and extensive sensor coverage. NIT technology, on the other hand, embeds electronic circuits directly into textile substrates using conductive fibers, yarns, or printed conductive inks. This approach creates flexible, stretchable, and lightweight circuits integrated across large fabric areas. Using weaving, embroidery, or layer-by-layer textile manufacturing, NIT circuits enable distributed sensing, actuation, and processing throughout the suit without compromising wearer comfort or mobility. Such textile-native circuits can host logic gates, sensors, and communication pathways within the fabric itself, allowing for real-time physiological monitoring, environmental sensing, and adaptive responses critical in aerospace settings. The flexibility and durability of NIT systems also improve resistance to mechanical strain, washing, and environmental factors compared to traditional electronics. By replacing bulky PCBs with integrated textile circuits, pilot suits can become more ergonomic and multifunctional. This integration technique supports the development of smart garments capable of autonomous operation and complex data management directly on the textile platform, which are key advances for the next generation of intelligent pilot suits.

Embedding miniature light-emitting diodes directly into conductive fibers enables lightweight, flexible lighting or signaling systems fully integrated into pilot suits. This approach allows real-time visual feedback such as status indicators, hazard warnings, or communication signals without adding bulk or restricting movement. The two-terminal connection design ensures simple, robust electrical interfacing, facilitating seamless integration with the suit’s electronic network. As shown in Figure 8c, LED yarns woven into flexible textile circuits can be combined with sensors and actuators to create multifunctional garments capable of responsive lighting based on physiological or environmental data [176]. Their flexibility and durability allow them to endure repeated stretching, bending, and environmental exposure typical of flight conditions. By embedding LED yarns directly into pilot suits, designers enhance situational awareness and pilot safety through intuitive, garment-integrated visual cues, representing an important advancement for next-generation aerospace wearables.

ASC fabric, composed of silver nanoparticles (AgNPs), styrene-butadiene-styrene (SBS), and Chinlon, offers a highly stretchable and conductive textile platform suitable for modern pilot suits. Its composite structure combines electrical conductivity with mechanical flexibility, enabling integration of sensors and circuits that can withstand body movement and flight-induced strain. As shown in Figure 8d, the fabrication scheme produces a fabric that maintains conductivity and structural integrity before and after stretching, confirmed by SEM images at various magnifications. This makes ASC fabric ideal for embedding functional electronics directly into garments, supporting real-time data transmission, body-conforming designs, and long-term reliability in aerospace environments [177].

### 4.2. Data Transmission and Communication Technologies

Advanced pilot suits increasingly rely on robust data transmission and communication technologies to enable real-time monitoring, control, and situational awareness [16,178]. These systems integrate wireless protocols such as Bluetooth Low Energy (BLE), Wi-Fi, and emerging 5G/6G networks to ensure low-latency, high-bandwidth connectivity between embedded sensors, actuators, and external systems [179]. Secure and reliable data channels are essential for transmitting physiological data, suit status, and environmental inputs to onboard aircraft systems or ground control. In addition, inter-device communication within the suit allows for adaptive responses, such as dynamic pressure regulation or thermal control. As pilot suits evolve toward greater autonomy and intelligence, the integration of decentralized communication architectures and edge computing further enhances responsiveness and reduces dependence on external infrastructure [180]. These technologies form a critical backbone for the functionality, safety, and performance of next-generation flight garments.

**Figure 9:**
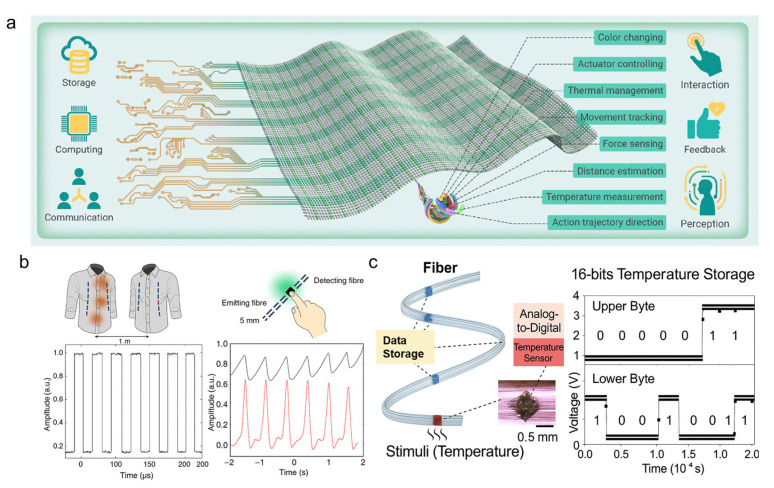
Textile-based data transmission and communication technologies for future pilot suits. (**a**) Multifunctional digital fibers woven into textiles to create a fabric computing platform capable of sensing, storing, and processing data. Individual memory elements within fibers can store up to 767 kb and maintain data for over 2 months without power [181]. (**b**) Fabric-based optical communication enabled by diode fibers integrating both light-emitting and photo detecting functions, achieving a 3 MHz bandwidth and operating across fabric distances up to 1 m [182]. Experimental results of the current measured by the photodetecting fiber (black curve) compared to the output of a commercial pulse sensor (red curve). (**c**) Hybrid digital fibers capable of temperature sensing, signal conversion, and storage; e.g., a digital fiber with 165 sensors and 767 kb memory operated across a 13 h test without data loss [183].

To be specific, multifunctional fiber and fabric technologies driven by multi-material fiber manufacturing are accelerating the development of intelligent pilot suits. These advanced textiles integrate digital-device-enabled fibers with embedded capabilities capable of sensing, data processing, signal transmission, and storage, forming a distributed computing platform directly within the fabric. Such systems enable real-time physiological monitoring, seamless human–machine interaction, and adaptive on-body learning. As illustrated in Figure 9a, this fabric computing paradigm supports a broad range of imperceptible yet powerful functions, including motion and trajectory tracking, force and distance sensing, temperature detection, color change, actuator control, and real-time feedback all embedded within the textile itself [181]. These features hold great potential for future aerospace garments requiring responsive, lightweight, and unobtrusive computing systems. These integrated systems support seamless communication between the suit, onboard aircraft systems, and external control centers, ensuring pilots receive timely feedback and enhanced situational awareness through reliable, responsive, and adaptive textile-based networks.

Recent studies have reported the successful integration of high-speed optical transmitters and receivers into textiles, enabling fabric-to-fabric communication and data transmission directly through the garment (Figure 9b) [182]. This advancement supports fabric-based light fidelity (Li-Fi) and encrypted local information transfer, technologies highly relevant to next-generation pilot suits. For example, light-detecting fibers have been fabricated using polycarbonate (PC) as the cladding material, embedded with silicon photodiodes, and integrated with InGaN and AlGaInP LEDs serving as optical transmitters. During the thermal drawing process, tungsten or copper wires are co-fed into the fiber structure to establish electrical connections with the devices. Upon light illumination, the photodetectors generate photo-induced charge carriers (electron–hole pairs), which are collected by the electrodes, enabling real-time conversion of optical signals into electrical outputs. These optical fibers embedded within garments can function as physiological sensors, capturing data such as heart rate through changes in light reflectance from the skin. This allows for continuous, non-invasive monitoring without bulky external devices. By integrating both communication and sensing directly into the fabric, these technologies transform the pilot suit into an intelligent, responsive platform capable of real-time data collection, processing, and transmission, which is key to supporting the cognitive and physical demands of modern aviation environments.

A novel digital fiber combining memory and temperature-sensing (MT) functions along a single strand is fabricated using a multi-material architecture. The fiber comprises a polycarbonate (PC) outer shell for mechanical protection, a soft PMMA (poly(methyl methacrylate)) inner layer that accommodates embedded devices, and tungsten wire electrodes for electrical interfacing. Integrated within this architecture are micro–nano optoelectronic components, including silicon-based temperature sensors and InGaN/AlGaInP semiconductor chips, which rely on nanoscale junctions for efficient electron–hole separation and signal transduction. These components are precisely positioned along the fiber axis via a CNC-milled PMMA preform and integrated using a thermal drawing process. Such nanoscale interfaces, combined with PC/PMMA confinement layers, ensure mechanical flexibility while maintaining high electrical and optical performance. This configuration enables local analog-to-digital conversion and on-fiber storage of temperature data, with 16 bit readings stored in embedded memory units, as shown in Figure 9c [183]. Unlike conventional fibers with uniform function, this design allows independently addressable sensing and memory nodes distributed along the fiber. Integrating this technology into pilot suits enables fast, reliable physiological monitoring with real-time data capture and storage directly at the textile level, improving communication efficiency between sensors and onboard systems, critical for flight safety and situational awareness.

### 4.3. Power Management and Energy Storage Solutions

Effective power management and energy storage are essential for modern pilot suits that integrate sensors, communication systems, and environmental monitors. These suits require compact, lightweight energy solutions that maintain flexibility and do not hinder mobility. Recent advances include lithium-ion and thin-film batteries [184,185], as well as fabric-integrated supercapacitors [186,187], which offer high energy density and can be seamlessly embedded into the suit. Smart power management systems ensure efficient energy distribution, voltage regulation, and load balancing across various components. To further enhance autonomy, energy harvesting technologies such as thermoelectric generators that convert body heat and piezoelectric materials that capture motion [188], are being explored to supplement battery power. Together, these technologies create a reliable and adaptive energy system that supports continuous operation of onboard electronics, even during extended missions. By combining efficient storage, intelligent distribution, and self-recharging capabilities, next-generation pilot suits are better equipped to ensure operational safety, situational awareness, and endurance in complex flight environments.

**Figure 10:**
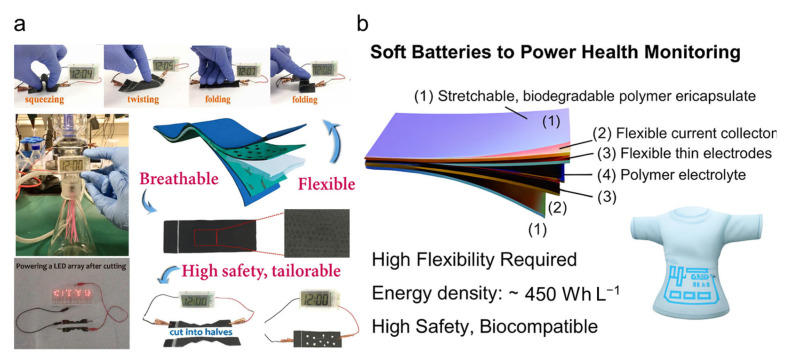
Power solutions for next-generation pilot suits. (**a**) Flexible, breathable quasi-solid-state lithium-ion battery with high safety and stability [190]. (**b**) Soft, skin-conformal bendable batteries for seamless integration and reliable power in wearable systems, reaching optimal energy density of 450 Wh L^−1^ [191].

Among emerging wearable power technologies, all-solid-state rechargeable lithium-ion batteries (ARLIBs) have gained attention for their enhanced safety, mechanical flexibility, and energy density qualities, which are especially critical for aerospace-grade smart suits [189]. Flexible and safe energy storage is essential for modern pilot suits, which must power integrated systems like sensors and communications without hindering mobility. The recent development of a quasi-solid-state aqueous ARLIB offers a strong solution, combining flexibility, breathability, and operational safety. Earlier studies explored thin-film batteries and fabric-based supercapacitors, but often faced trade-offs between durability, performance, and wearer comfort [190]. This ARLIB, built on carbon cloth with a gel polymer electrolyte, withstands bending, twisting, and folding, making it ideal for high-mobility flight conditions (Figure 10a). Uniquely, it can be shaped or perforated to enhance breathability, which is an important comfort factor in wearable systems. These features directly support the integration of energy storage into next-generation pilot suits, offering a lightweight, safe, and conformable power solution for sustained and reliable performance during demanding missions.

In addition, several studies reveal that wearable batteries vary widely in flexibility and energy density. Recent research highlights the importance of classifying batteries into unidirectional (bendable) and omnidirectional (soft, deformable) types to better match application requirements [191]. This distinction helps optimize battery integration in pilot suits featuring bendable batteries suit structured areas, while soft, conformable ones fit close-contact zones for health monitoring (Figure 10b). Clearer categorization and targeted design improve power management by enhancing comfort, durability, and performance, addressing key challenges in wearable energy storage for aerospace applications. Furthermore, emerging studies are beginning to explore the hybridization of flexible energy storage with triboelectric or thermoelectric energy harvesting modules, aiming to establish autonomous, self-sustaining power systems for aerospace wearables [192].

## 5. Limitations, Challenges, and Prospects

While the technological trajectory of smart pilot suits is both compelling and evolving, it is important to acknowledge that regulatory compliance, long-term safety validation, and durability testing remain significant challenges before widespread aerospace deployment can be realized. Many of the advanced materials and integrated systems discussed in this review, though promising at the laboratory and prototype level, mostly have not yet undergone rigorous qualification under aviation standards. As such, future progress will depend not only on innovations in materials and electronics, but also on the establishment of robust certification pathways and flight-grade validation protocols. Collaborative efforts among materials scientists, aerospace engineers, and regulatory agencies will be crucial to bridging this gap, accelerating the transition from experimental innovation to certified operational systems.

Future developments will focus on trendy material intelligence [193,194], miniaturized neuromorphic computing [195], and fully integrated data-driven sensing within the textile matrix [196,197,198]. Smart polymer composites capable of dynamic response, self-healing, or reconfiguration under extreme operational conditions will further expand applications. The convergence of advanced polymers, AI-enabled electronics, and human-centric design is poised to redefine the role of the pilot suit from passive equipment to an active, responsive platform that augments physiological resilience and cognitive performance. Continued interdisciplinary collaboration will be essential to realize these capabilities and ensure robust performance in next-generation aerospace missions.

## 6. Conclusions

Innovations in fabrication strategies including fiber spinning, 3D/4D printing, and multilayer integration (e.g., nanoimprinting, electrospinning, and layer-by-layer assembly) have expanded the design space for pilot suit architectures at both micro- and nanoscale levels. These approaches, combined with the integration of high-performance fibers, nanomaterials, and multifunctional textile systems, are driving the transition from passive protection to adaptive and responsive garments. Additionally, recent progress in flexible electronics and fiber-based sensing technologies enables real-time physiological and environmental monitoring within textile platforms.

Despite these advancements, large-scale aerospace deployment remains highly challenging. Issues such as material durability under extreme environments, energy supply for embedded systems, and compliance with stringent aviation certification standards require systematic solutions. Future developments are likely to leverage nanomaterials, particularly through hierarchical structuring, surface functionalization, and hybrid composites, to enable multifunctional properties such as thermal adaptability, electromagnetic shielding, energy storage, and integrated sensing within a single textile platform. The convergence of these capabilities with scalable fabrication will be critical to achieving truly autonomous and adaptive pilot suits. Bridging this gap will demand continued interdisciplinary collaboration among materials scientists, aerospace engineers, and regulatory authorities. Addressing these challenges will be critical for translating current laboratory innovations into certified, mission-ready systems that meet the operational demands of next-generation aviation textiles and devices.

## Figures and Tables

**Table 1:** Functional nanomaterials for advanced textile applications in pilot suits.

Nanomaterial	Key Functional Properties	Integration Strategy	Relevant Applications	Refs.
Carbon nanotubes	High tensile strength, conductivity, piezoresistivity	Wet-spinning into fiber yarns or coatings	Impact buffering, strain sensing	[80]
Graphene	Flexibility, EMI shielding, thermal conductivity	Layered lamination, inkjet printing	Data transmission, thermal regulation	[81]
MXenes	High conductivity, EMI shielding, redox-active sites	Dip-coating on fabrics, fiber composite	Energy storage, EMI shielding	[82]
Nanoclusters (e.g., Ag)	Catalytic reactivity, fluorescence, sensing	Surface grafting, sol–gel deposition	Biosignal sensing, wound detection	[83]
MOFs	Porosity, chemical tunability, gas interaction	Electrospun blends, particle embedding	Breathability, gas sensing, heat exchange	[84]

**Table 2:** Performances and applications of engineering fibers in pilot suit.

Fiber Type	Density (g/cm^3^)	Key Advantages	Limitations	Typical Application	Refs.
Aramid (Kevlar^®^/Twaron^®^)	~1.44	High strength-to-weight ratio, flexibility, flame resistance, ballistic protection	Moderate UV degradation, limited thermal resistance above 300 °C	Body armor, joint reinforcement, flame-resistant layers	[85,86]
PBO (Zylon^®^)	~1.54	Extreme heat resistance, highest LOI among organic fibers, high tensile strength	Susceptible to photodegradation, requires UV-blocking treatments	Outer layers for high heat exposure (e.g., ejection systems)	[92,93]
Polyimide (P84^®^)	~1.41	Broad temperature tolerance, inherent flame resistance, chemical inertness	Lower mechanical strength compared to aramids or PBO	Thermal insulation, moisture-wicking liners	[88,89]
Carbon Fiber	1.75–2.0	High stiffness, impact dispersion, thermal barrier properties	Brittle under shear stress, higher cost, requires resin matrices	Structural reinforcement, heat shields	[94]
UHMWPE	~0.97	Lightest commercial fiber, chemical resistance	Low melting point, flame retardant	Lightweight limb protection, anti-abrasion	[95]

**Table 3:** Quantitative energy output, integration strategies, and application targets of triboelectric fibers under representative pilot motion scenarios.

Motion Scenario	Avg. Output (μW/cm^2^)	Peak Output (μW/cm^2^)	Integration Strategy	Application Target	Refs.
Arm motion (control stick operation)	4.5	7	Embedded in sleeve seams using conductive embroidery; direct connection to forearm sensor clusters	Vital sign sensors (heart rate, muscle activity)	[117,118]
Leg movement (pedals/ejection prep)	10.8	14	Knitted into elastic compression layers; routed to modules above ankle or knee	Local thermal control units; joint strain monitors	[119]
Torso rotation (instrument scanning)	3.5	6	Laminated onto mesh fabrics around the waist; anchored at low-strain zones	Core temperature regulation; posture monitoring system	[117]
Micro-movements under gravitational force load	0.9	1.5	Braided into inner suit lining; coupled with capacitors in spinal region	Backup energy buffering; low-power fatigue sensors	[120]
Neck/head tilt (helmet enclosure)	50	316	Woven into collar fabric; ultrathin leads connect to helmet rear module	Oxygen saturation sensors; ambient temperature sensors	[121,122]

**Table 4:** Summary of 4D-printable polymers for integration into smart textiles in modern pilot suits.

Type	Examples	Stimuli Response	Advantages for Pilot Suit	Textile Integration	Printable Process Compatibility	Mechanical Properties	Refs.
Shape Memory Polymers (SMP)	Polyurethane SMP, epoxy SMP, crosslinked PCL	Heat (thermal triggers)	Adaptive fit, impact and thermal protection	Requires tuning transition temp; compatible with FDM, DIW	FDM, DIW, SLA (formulated resins)	Tensile strength: 10–50 MPa; elongation: 50–300%	[153,154]
Thermoresponsive Polymers	PNIPAM, block copolymers	Temperature (LCST ~32 °C)	Breathability control, thermal comfort	Often hydrogels needing blending for durability	DIW, SLA (hydrogel resins)	Tensile strength: 1–10 MPa; elongation: 100–500%	[155,156]
Photoresponsive Polymers	Azobenzene-based, spiropyran-modified	UV/Visible light	Remote, localized activation; stiffness control	UV exposure control needed; integrates with photopolymer resins	SLA, DLP, PolyJet	Tensile strength: 10–40 MPa; elongation: 20–200%	[157]
Electroactive Polymers	Polypyrrole, polyaniline, dielectric elastomers	Electrical stimulus	Active shape change, ventilation, fit adjust	Needs conductive fillers/electrodes; complex integration	DIW, PolyJet	Tensile strength: 5–30 MPa; elongation: 30–200%	[158]
Strain-Hardening Polymers	TPU with additives	Mechanical stress	Impact protection; stiffening under load	Compatible with extrusion and ink-based printing	FDM, DIW	Tensile strength: 20–60 MPa; elongation: 200–600%	[159,160]
Hydrogels	Polyacrylamide, alginate-based	Moisture, humidity	Moisture management, swelling actuation	Needs reinforcement for textile strength	DIW, SLA	Tensile strength: 0.1–5 MPa; elongation: 200–1000%	[161,162]
Self-Healing Polymers	Diels–Alder crosslinked, hydrogen-bond elastomers	Heat, moisture, light	Self-repair, extended lifespan	Emerging tech, challenging integration	DIW, SLA (specialized formulations)	Tensile strength: 5–30 MPa; elongation: 50–300%	[163,164]

**Table 5:** Representative actuation mechanisms and potential integration in pilot suit systems.

Actuation Type	Working Mechanism	Potential Functions in Pilot Suit	Aerospace Suit Application Scenario	Refs.
Electrothermal actuators	Thermal expansion via resistive heating	Localized heating or compression adjustment	High-altitude temperature drops	[165]
Pneumatic actuators	Air pressure inflation/deflation	Ventilation and cooling control	Rapid cockpit pressure changes	[166]
Hydrogel-based actuators	Swelling/deswelling to temperature or moisture change	Moisture-responsive fit modulation	Sweating/thermal stress under tight suits	[167]
Shape memory alloys (SMA)	Phase change-induced deformation	Structural adaptation or joint support	Dynamic posture correction	[168]
Electroactive polymers (EAP)	Reversible deformation under electric field	Muscle-assist or tactile feedback during gravitational forces	Autonomous support during gravitational force exposure	[169]

## Data Availability

All the data are presented within this review article.

## Abbreviations

**Full Name**	**Abbreviation**	**Full Name**	**Abbreviation**
Thermoplastic polyurethane	TPU	Lower critical solution temperature	LCST
Triboelectric nanogenerator	TENG	Poly(N-isopropylacrylamide)	PNIPAM
Carbon nanotubes	CNTs	Poly(p-phenylene-2,6-benzobisoxazole	PBO
Limiting oxygen index	LOI	Polyacrylamide	PAAm
Shape memory polymers	SMP	Polypyrrole	PPy
Metal–organic frameworks	MOFs	Stereolithography	SLA
Direct ink writing	DIW	Digital light processing	DLP
Polyimide	PI	Polyaniline	PANI
Polyimide (commercial grade, P84^®^)	P84^®^	Artificial Intelligence	AI
Polyethylene Glycol	PEG	Fused Deposition Modeling	FDM
Electromagnetic Interference Shielding	EMI	poly(methyl methacrylate	PMMA
